# Adipocyte Enhancer Binding Protein 1 (AEBP1) Inhibition as a Potential Anti-Fibrotic Therapy in Heart Failure

**DOI:** 10.21203/rs.3.rs-3390276/v2

**Published:** 2026-05-26

**Authors:** Thirupura Sundari Shankar, Joseph Visker, Junedh Amrute, Georgiy Polishchuk, Ty Lunde, Jing Ling, Peter Ferrin, Rana Hamouche, Dominik Feigle, Dallen Calder, Frank Sachse, Christos Kyriakopoulos, Eleni Maneta, Eleni Tseliou, Sutip Navankasattusas, Craig Selzman, Thomas Seidel, Kory Lavine, Stavros Drakos

**Affiliations:** University of Utah; University of Utah; Washington University School of Medicine; University of Utah; University of Utah; University of Utah; University of Utah; The University of Utah; Friedrich-Alexander University (FAU); University of Utah; University of Utah; University of Utah School of Medicine; University of Utah; University of Utah; University of Utah; University of Utah; Friedrich-Alexander University Erlangen-Nürnberg (FAU); Washington University School of Medicine; U Utah

## Abstract

Persistent activation of cardiac fibroblasts into myofibroblasts drives excessive extracellular matrix deposition, leading to maladaptive myocardial fibrosis, adverse remodeling and heart failure (HF) progression. Adipocyte enhancer binding protein 1 (AEBP1) and Aortic carboxypeptidase-like protein (ACLP, full-length AEBP1 protein coded by *AEBP1*) has been implicated in pathological fibrosis across multiple organs, with tissue-specific knockdown attenuating fibrosis in preclinical models. Although elevated *AEBP1* expression has been associated with human HF, its role in myocardial fibrosis progression in vivo remains undefined. Here, we demonstrated that *AEBP1* is a critical mediator of myocardial fibrosis and adverse cardiac remodeling. Fibroblast-specific knockout and cardiac-specific knockdown of *Aebp1* significantly improved cardiac function and prevented pathological remodeling in murine models of myocardial ischemia and pressure-overload induced injury. In ex vivo human myocardial tissue culture studies, ACLP overexpression in non-failing hearts induced pathological remodeling, whereas *AEBP1* knockdown in failing human hearts induced structural reverse remodeling. Mechanistically, we also showed that ACLP regulates key pro-fibrotic transcription factors and genes, including MRTFB, RUNX2, SM22 and COL1A1, thereby orchestrating fibroblast activation. Collectively, these findings establish *AEBP1* as a central driver of myocardial fibrosis and highlights its inhibition as a promising therapeutic strategy to mitigate both acute and chronic HF.

## Introduction

Increased myocardial fibrosis is one of the hallmarks of cardiac remodeling observed in chronic heart failure (HF) patients^1–3^. Quiescent fibroblasts in the heart are activated to form myofibroblasts as a response to stress or injury including hypertension, inflammation, and myocardial infarction (MI). These myofibroblasts secrete extracellular matrix (ECM) components, mainly collagen, which replaces the injured and necrotic myocytes. While this initial fibrotic response helps prevent cardiac rupture, continued fibroblast activation and myofibroblast proliferation, leads to excess ECM deposition and drives adverse cardiac remodeling and HF progression^4–6^.

Identifying common genes and networks in multi-organ fibrosis has been a target of intense investigation^7,8^. Recent studies have identified adipocyte enhancer binding protein 1 (*AEBP1*) and aortic carboxypeptidase-like protein (ACLP, a full-length protein coded by *AEBP1*) as a crucial activator of fibroblasts in cardiac, hepatic, pulmonary, adipose tissue and renal fibrosis^9–12^. ACLP was shown to activate fibroblasts leading to increased fibrosis, whereas *Aebp1* knock-down (KD) in animal models of organ fibrosis attenuated hepatic, pulmonary and renal fibroblast activation^9,10,12^. Recent studies also showed increased *AEBP1* gene expression to be associated with inflammation and fibrosis in HF^13^ and *AEBP1* KD in human cardiac fibroblasts (HCF) prevented its differentiation to myofibroblast^14^. However, the role and mechanism of ACLP and the N-terminal truncated version of ACLP (referred to as AEBP1) encoded by the same gene in mediating cardiac fibroblast activation in vivo is not well understood.

ACLP is also known to be involved in the wound-healing process. Whole body *Aebp1* developmental knock-out (KO) mice exhibit abnormal wound healing and reduced fibroblast proliferation^15^. Bi-allelic pathogenic variants of *AEBP1* were found in patients with Ehlers Danlos Syndrome (EDS), a rare inherited disorder. The affected individuals have defective collagen polymerization leading to stretchy and fragile skin and hypermobility of the joints^16^. On the contrary, *AEBP1* overexpression (OE) has been associated with increased risk for abdominal aortic aneurysms, Alzheimer’s disease, breast cancer, glioblastoma and several other cancers^17–20^ in addition to the aforementioned cardiac, hepatic, pulmonary and renal fibrosis^9,10,21^.

The overall goal of this study is to understand the role of *Aebp1* inhibition in mice models of HF and fibrosis. We hypothesized that ACLP primarily drives cardiac fibroblast activation and *Aebp1* knockout (KO) following pressure overload or MI prevents persistent fibroblast activation and could lead to reduced fibrosis and improved cardiac function. To test our hypothesis, we used acute and chronic HF mouse models where *Aebp1* expression was knocked out at different timepoints. In summary, our data showed that, irrespective of the time of intervention, *Aebp1* KO in mouse models of acute MI, was beneficial, prevented HF progression and improved cardiac function. *AEBP1* KD in chronic human HF myocardium, resulted in a significant reduction in myocardial fibrosis and an improvement in myocardial architecture compared to paired untreated control. We demonstrated the role of *Aebp1* inhibition in preventing fibroblast activation in vivo and promoting reverse cardiac remodeling. In addition, we also showed that ACLP regulates the expression of major pro-fibrotic transcription factors and genes. *Aebp1* inhibition suppressed fibroblast activation by inhibiting pro-fibrotic targets including MRTFB, RUNX2, COL1A1, SM22, and other regulators of fibrosis. Overall, our results show a unique regulation of fibroblast activation via ACLP indicating that AEBP1/ACLP inhibition could be a potential anti-fibrotic target in acute and chronic HF.

## Results

### Increased expression of *AEBP1* is an indicator of pathological remodeling in the heart.

**Figure 1:**
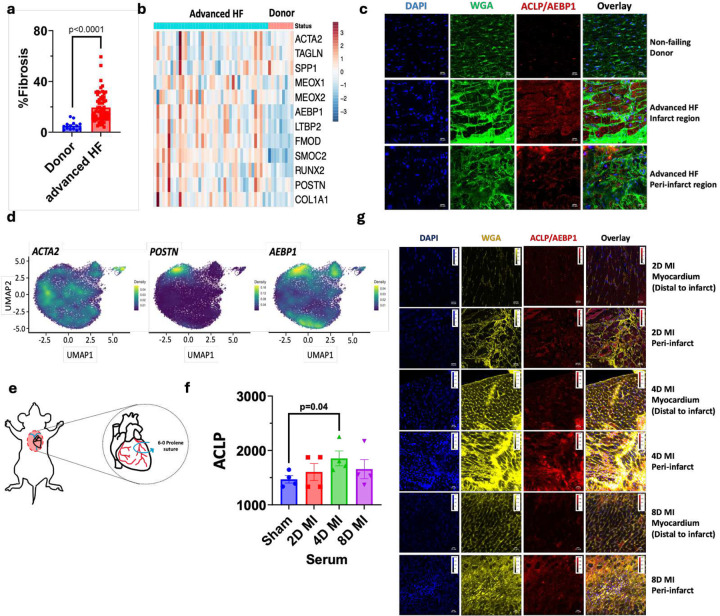
Increased expression of *AEBP1* is an indicator of pathological remodeling in the heart. **a,** Masson trichrome analysis of human non-failing donor and advanced HF myocardium (n=14 donor, n=61 HF). **b,** Differential expression analysis between donor and advanced HF myocardium (n=9 donor, n=41 HF). **c,** Representative immunohistochemistry image of non-failing myocardium and infarct and peri-infarct regions of human HF myocardium (n=3). **d,** UMAP of fibroblast cluster showing the topology of *ACTA2, POSTN* and *AEBP1* expression in HF compared to donor fibroblasts respectively (n=14 donor and n=13 HF). **e,** Mouse myocardial infarction (MI) model depiction. **f,** Serum ELISA quantifying ACLP levels (n=4 each). **g,** Representative immunohistochemistry of mouse infarct/peri-infarct and distal myocardium at 2-, 4- and 8-days post MI (n=4 each). p-value: unpaired t-test (panel a), One-way ANOVA (panel f).

RNA sequencing on myocardial tissue acquired from advanced HF patients with elevated myocardial fibrosis showed a significant increase in *AEBP1* transcript levels along with a subsequent increase in well-known pro-fibrotic transcription factors (including *RUNX1*^22^*, RUNX2*^23^ and *MEOX1*^24^) and pro-fibrotic genes (including *ACTA2*^25^, *TAGLN*^26^, *SMOC2*^27^ and *COL1A1*) (Fig. 1a–b). Ingenuity pathway analysis^28^ demonstrated that pathways implicated in fibrosis, cardiac hypertrophy and mitochondrial dysfunction were significantly upregulated in the fibrotic HF myocardium compared to non-failing donor myocardium (Supplemental Fig. 1a–f). Immunohistochemistry data revealed increased localization of ACLP/AEBP1 in the ECM of infarct and peri-infarct regions of advanced HF myocardium with little to no expression in the non-failing myocardium (Fig. 1c). ACLP/AEBP1 was expressed in multiple cell types and in the ECM of myocardial tissue from chronic HF patients. Despite being expressed at low level in other cell types, single-nuclear RNA sequencing (snRNA seq) data showed a significant increase in *AEBP1* expression in fibroblasts over other cell types (Supplemental Fig. 2a–b). We observed increased localization of *AEBP1* in activated fibroblasts that also had increased expression of *ACTA2* (α-smooth muscle actin) and *POSTN* (periostin) (Fig. 1d). To better understand the mechanistic role of *Aebp1* in pathological remodeling, we induced MI in mice by ligating left anterior descending coronary artery (Fig. 1e) and observed a significant increase in circulating ACLP acutely following MI (4 days) compared to sham (Fig. 1f). Immunohistochemistry data again showed increased localization of ACLP/AEBP1 in infarct and peri-infarct regions and little to no expression in distal myocardium or in myocardium of sham mice (Fig. 1g). Immunohistochemistry data again confirmed ACLP/AEBP1 localization to fibroblasts in mouse myocardium (Supplemental Fig. 2c), indicating *Aebp1* expression to be specific to activated fibroblasts and increased expression of ACLP/AEBP1 near peri-infarct region maybe associated with pathological remodeling and fibrosis expansion.

### *Aebp1* inhibition improves cardiac function in mice with MI.

**Figure 2:**
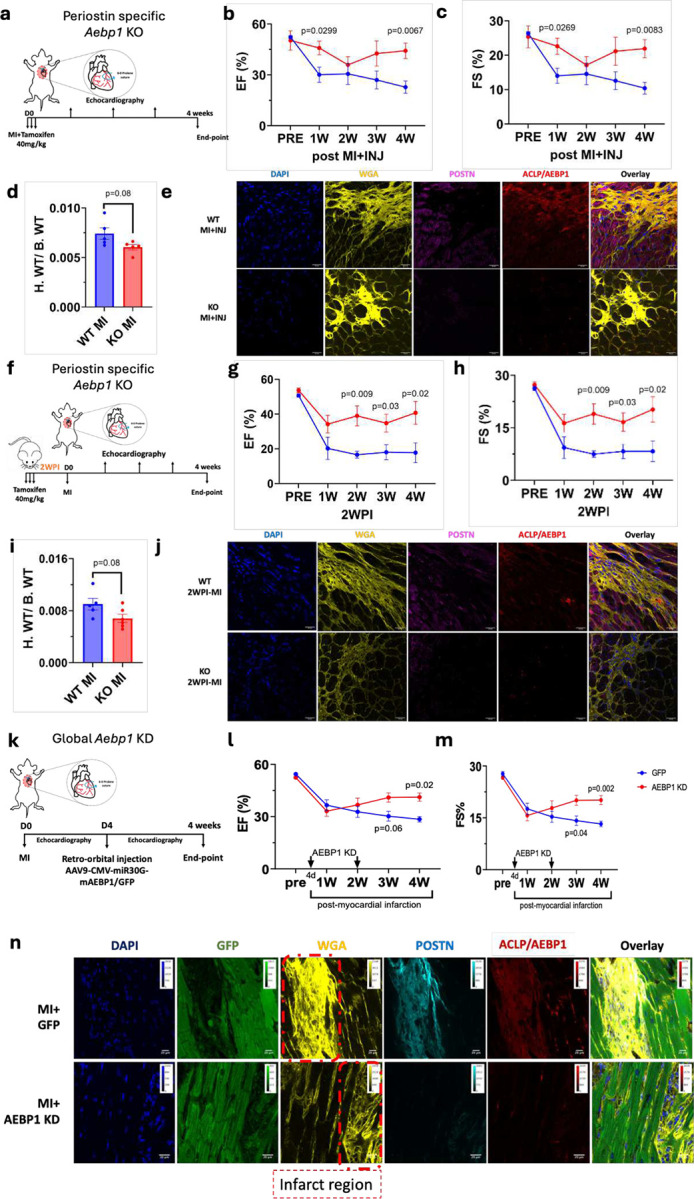
*Aebp1* inhibition improves cardiac function in mice with MI. **a,** Schematic of fibroblast-specific *Aebp1* KO along with MI induction (MI+inj). **b-c,** Left ventricular ejection fraction (EF%) and fractional shortening (FS%) data in MI+inj group (n=5 each) **d,** Heart weight to body weight ratio in MI+inj group (n=5 each). **e,** Representative immunohistochemistry data in WT and fibroblast-specific *Aebp1* KO (n=5 each). **f,** Schematic of fibroblast-specific *Aebp1* KO 2 weeks post tamoxifen injection (2wpi-MI). **g-h,** EF% and FS% data in 2wpi-MI (n=5 each) **i,** Heart weight to body weight ratio in WT and KO mice (n=5 each). **j,** Representative immunohistochemistry data in WT and fibroblast-specific *Aebp1* KO 2wpi (n=5 each). **k,** Schematic of global *Aebp1* KD using AAV9 specifically in the heart. **l-m,** EF% and FS% data following *Aebp1* KD (n=5 each). **n,** Representative immunohistochemistry data in WT GFP and *Aebp1* KD mice (n=5 each). p-value: One way ANOVA (panels b-c, g-h and l-m), unpaired t-test (panels d&i).

Given the specificity of *AEBP1* to activated fibroblasts (Supplemental Fig. 2a–c), we used a tamoxifen inducible periostin-specific promoter to knockout *Aebp1* in mice^29^. We used mouse models in which *Aebp1* was knocked out at three distinct timepoints relative to MI injury. Given *Aebp1’s* role in wound healing, we first did a simultaneous tamoxifen injection along with MI (KO/MI) to let the initial reparative fibrosis develop before we introduced the genetic deletion. Despite the presence of replacement fibrosis in *Aebp1* KO mice, we observed a significant improvement in cardiac function with higher ejection fraction (EF) and fraction shortening (FS) 4-weeks post-MI compared to wild-type (WT) with MI (Fig. 2a–c). We also observed a trend towards reduced cardiac hypertrophy as shown by decrease in heart weight/body weight ratio and reduced fibrosis expansion evidenced by reduced periostin positive cells in *Aebp1* KO mice (Fig. 2d and e). Second, we performed MI in mice 2 weeks post tamoxifen injection to assess if *Aebp1* KO mice may be susceptible to cardiac rupture. The *Aebp1* KO mice showed a significant improvement in cardiac function as early as 2-weeks post-MI compared to WT MI with a trend towards reduced cardiac hypertrophy and reduced periostin positive cells in KO (Fig. 2f–j). Third, we knocked out *Aebp1* after the mice developed HF with left ventricular ejection fraction (EF)<35% to assess if *Aebp1* KO can reverse the HF phenotype. We observed that the KO mice showed an improvement in function 6 weeks post-MI; however, it was not significant indicating the need for either a longer duration of follow up or an earlier intervention in order to observe significant cardiac recovery (Supplemental Fig, 3a–d). Overall, our data indicate the anti-fibrotic role of AEBP1 KO in this acute MI model.

Finally, to test for systemic toxicity and the therapeutic potential of *Aebp1* inhibition, we used a AAV9 viral vector with CMV driven siRNA (AAV9-CMV-miR30G-m*Aebp*1) that will induce *Aebp1* KD in all cells that express the gene. We performed retro-orbital injection 4 days post-MI and observed no apparent systemic toxicity and a significant improvement in cardiac function 4 weeks post-MI (Fig. 2k–m), again reiterating the beneficial effects of ACLP/AEBP1 inhibition following MI. Immunohistochemistry data showed a significant reduction in periostin positive cells around the infarct region in *Aebp1* KD mice compared to untreated WT mice indicating a potential inhibition of fibroblast activation following ACLP/AEBP1 inhibition (Fig, 2n).

### *Aebp1* inhibition suppresses interstitial fibrosis progression and cardiac hypertrophy in mice with pressure overload.

**Figure 3:**
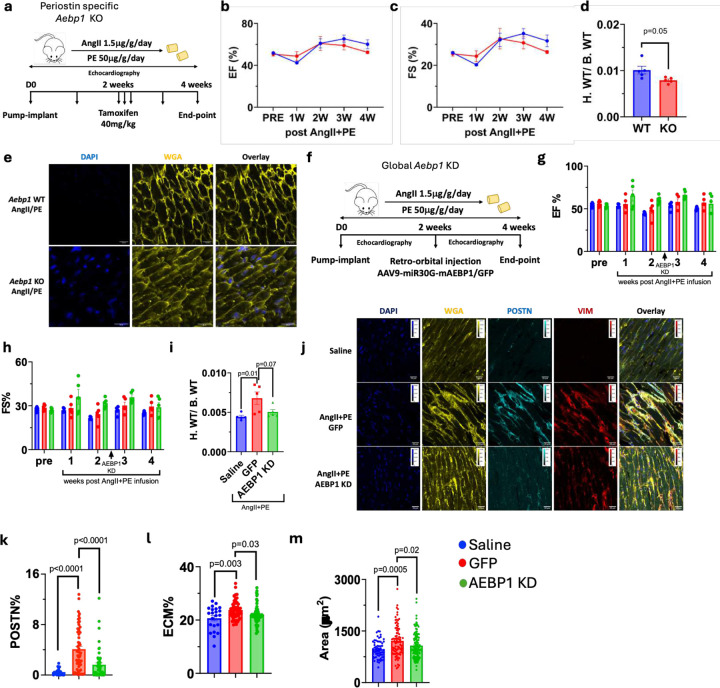
*Aebp1* inhibition suppresses interstitial fibrosis progression and cardiac hypertrophy in mice with pressure overload. **a,** Schematic of AngII/PE infusion and fibroblast-specific *Aebp1* KO. **b-c,** Left ventricular ejection fraction (EF%) and fractional shortening (FS%) data (n=5 each). **d,** Heart weight to body weight ratio (n=5 each). **e,** Representative immunohistochemistry of WT and KO myocardium following AngII/PE infusion (n=5 each). **f,** Schematic of AngII/PE infusion and global *Aebp1* KD. **g-h,** EF% and FS% data (n=5 each). **i,** Heart weight to body weight ratio (n=4 saline, n=5 each of GFP and AEBP1 KD). **j,** Representative immunohistochemistry of mouse myocardium (n=4 saline, n=5 each GFP and AEBP1 KD). **k-m,** Quantification of periostin signal, ECM percentage measured from WGA signal intensity and cell surface area. p-value: Two-way ANOVA (panel b-c, g-h); unpaired t-test (panel d); One-way ANOVA (panel i, k, l and m).

Diffuse interstitial fibrosis has a greater potential for reversal compared to replacement fibrosis^30^. To determine the role of ACLP/AEBP1 in interstitial fibrosis, we knocked out *Aebp1* specifically in fibroblasts, 2 weeks after AngII/PE infusion (Fig. 3a). We observed no significant difference in cardiac function between *Aebp1* KO and WT (Fig. 3b–c). However, we observed a trend towards reduced cardiac hypertrophy as indicated by the decrease in heart weight/body weight ratio (Fig. 3d) and a distinct reduction in interstitial fibrosis as shown by reduction of interstitial extracellular matrix (ECM) stained by wheat germ agglutinin (WGA) in *Aebp1* KO mice compared to WT (Fig. 3e). Additionally, we also performed global cardiac *Aebp1* KD using siRNA (AAV9-CMV-miR30G-m*Aebp1*) in C57BL/6 mice following 2 weeks of combination drug infusion and observed similar results where no change in cardiac function was observed across all 3 groups (saline, untreated GFP control mice with AngII/PE infusion and *Aebp1* KD with AngII/PE infusion) (Fig, 3f–h). However, we observed reduced cardiac hypertrophy in *Aebp1* KD mice (Fig, 3i). Immunohistochemistry data showed disperse interstitial fibrosis in both groups of mice that received the combination drugs, however, we observed a significant reduction in periostin positive cells (suggesting reduced fibrosis expansion) following ACLP/AEBP1 suppression. Quantitative analysis showed a significant reduction in fibrosis [measured using mean WGA intensity outside the cell (ECM%)] and reduced cardiomyocyte hypertrophy, shown as a reduction in cardiomyocyte cell surface area in *Aebp1* KD mice compared to WT (Fig, 3j–m). We also showed the reduction of fibrosis expansion and improved cardiomyocyte structure using fibroblast-specific AAV9-Postn-miR30G-m*Aebp1* construct (Supplemental Fig, 4a–f). Overall, our data indicate that AEBP1 inhibition played a crucial role in preventing fibrosis expansion, thereby contributing to improved myocardial architecture following pressure overload.

### *AEBP1* plays a crucial role in cardiac fibrosis and cardiomyocyte remodeling in advanced HF.

**Figure 4:**
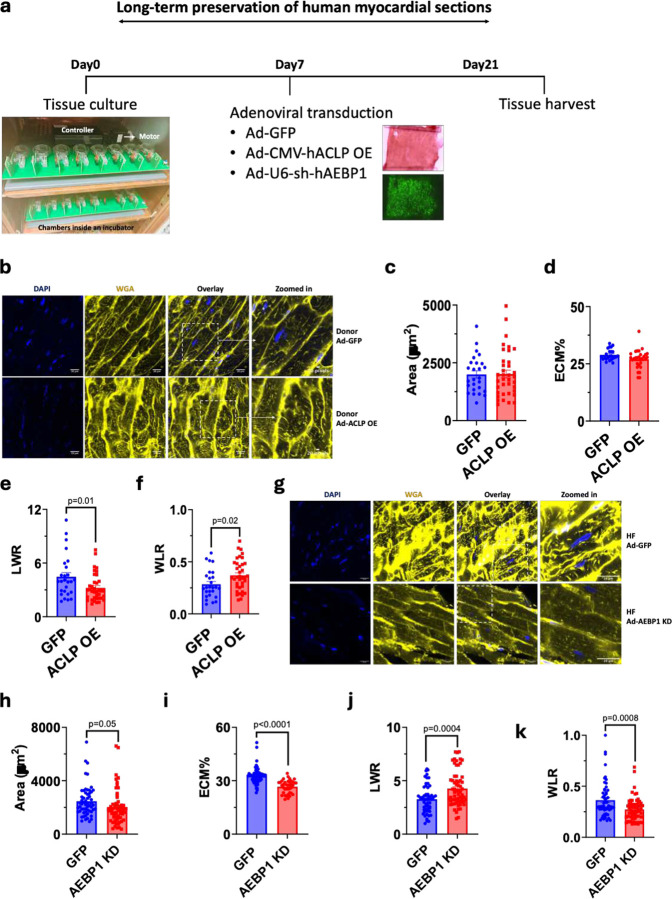
*AEBP1* plays a crucial role in cardiac fibrosis and cardiomyocyte remodeling in advanced human HF. **a,** Human myocardial tissue culture methodology. **b,** Representative immunohistochemistry data of cultured non-failing human myocardium (n=3). **c,** Quantification of cell surface area. **d,** ECM percentage measured from WGA signal intensity (n=3 donors, multiple images/donor). **e-f,** cell length to width ratio (LWR) and width to length ratio (WLR) from immunohistochemistry (n=3 donors, multiple images/donor). **g,** Representative immunohistochemistry data of cultured human HF myocardium (n=6). **h,** Quantification of cell surface area, **i,** ECM percentage measured from WGA signal intensity. **j-k,** LWR and WLR from immunohistochemistry (n=6 patients, multiple images/patient). p-value: unpaired t-test.

Although in response to acute cardiac injury, activation of fibroblasts serves to preserve the structural integrity of the ventricle, prolonged activation of fibroblasts may lead to excessive production and cross-linking of the ECM. Recent evidence also suggests that fibroblasts harvested from advanced HF patients exhibited a myofibroblast phenotype and expressed high levels of matricellular proteins and ECM cross-linking proteins^31,32^. Due to the absence of robust animal models to study biological mechanisms of fibrosis progression and regression in chronic HF and to confirm the role of ACLP/AEBP1 in human myocardium, we turned to long-term myocardial tissue culture of cardiac slices procured from non-failing and advanced HF patients (Fig. 4a). Briefly, myocardial slices were mounted onto the chambers and electrically paced at a constant rate to maintain tissue structure and function^33^. Advanced HF and non-failing myocardial slices were cultured and treated with adenovirus containing GFP, full-length *AEBP1* OE (ACLP OE), or *AEBP1* KD vector containing shRNA driven by U6 promoter. Paired samples from the same individual were used for all our comparisons. Despite unchanged cell surface area and fibrosis (no change in ECM%) (Fig. 4b–d), ACLP OE in non-failing donor myocardium resulted in structural remodeling evident from the decrease in cell length-width ratio (LWR) and increase in width-length ratio (WLR) indicative of cardiomyocyte remodeling compared to the GFP control (Fig. 4e–f)^34^. In contrast, *AEBP1* KD in human HF myocardium resulted in a significant reduction in cell surface area, a significant reduction in myocardial fibrosis, and an improvement in myocardial architecture. The LWR of myocardial cells following *AEBP1* KD increased, and WLR decreased compared to GFP control slices, suggesting more longitudinal and narrower cellular geometry similar to non-failing myocardium (Fig. 4g–k) and reiterating the importance of ACLP/AEBP1 inhibition in improving cardiac architecture in advanced HF myocardium.

### Increased ACLP drives cardiac fibrosis by regulating multiple pro-fibrotic targets.

**Figure 5:**
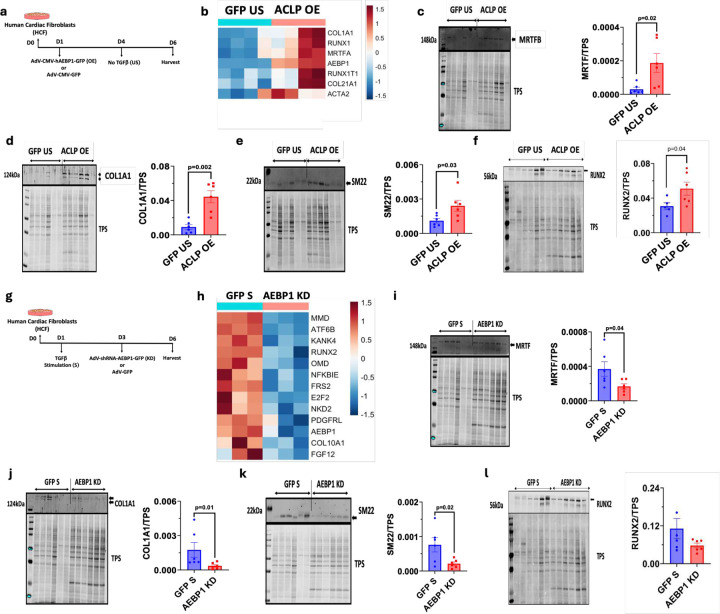
Increased ACLP drives cardiac fibrosis by regulating multiple pro-fibrotic targets. **a,** Schematic of primary human cardiac fibroblast (HCF) culture and ACLP overexpression (OE). **b,** Heatmap of GFP US and ACLP OE HCF (n=4 each). **c-f,** Western blot image and quantification of MRTFB, COL1A1, SM22 and RUNX2 in GFP US and ACLP OE (n=6 each for all targets, RUNX2 – n=5 GFP US and n=6 ACLP OE). **g,** Schematic of primary human cardiac fibroblast culture and *AEBP1* knockdown (KD). **h,** Heatmap of GFP S and AEBP1 KD HCF (n=3 each). **i-l,** Western blot image and quantification of MRTFB, COL1A1, SM22 and RUNX2 in GFP S and *AEBP1* KD (n=6 each for all targets, RUNX2 – n=5 GFP US and n=6 ACLP OE). p-value: unpaired t-test.

ACLP is a secreted protein implicated in the development of organ fibrosis. Previous studies have shown that ACLP activates fibroblasts via distinct signaling pathways, including TGF-β signaling, MRTFA, MAPK, NF-κβ, Akt and β-catenin^10,35,36^. To understand the mechanism behind ACLP-mediated cardiac fibroblast activation, we overexpressed ACLP in primary human cardiac fibroblasts (HCF) with adenovirus containing CMV that overexpresses full-length *AEBP1* (AdV-CMV-h*AEBP1*). Consistent with prior studies^14,37^, Ingenuity pathway analysis of the RNA sequencing data, showed a significant activation of fibrotic signaling, cell proliferation and mitochondrial dysfunction following ACLP overexpression, and a significant suppression of oxidative phosphorylation, sirtuin signaling and PKA signaling (Supplemental Fig. 5a–f). These findings indicated activation of fibrogenesis following ACLP expression compared to dormant fibroblasts. We observed a significant upregulation of major pro-fibrotic transcription factors and genes including *RUNX1, MEOX1, MRTFA, MRTFB, RUNX2, COL1A1, ACTA2* and *TAGLN* following ACLP OE, some of which were confirmed by western blot (Fig. 5a–f). Concurrently, we inhibited *AEBP1* expression in TGFb activated fibroblasts using shRNA (AdV-U6-shRNA-h*AEBP1*) and observed significant activation of PKA signaling, wound healing pathways and eNOS signaling following *AEBP1* KD compared to TGFβ-activated fibroblasts. *AEBP1* KD also resulted in a significant suppression of PD-1/PD-L1 signaling, IL-12, IL-10 and endothelin-1 signaling thereby suppressed inflammatory response and attenuated fibroblast activation (Supplemental Fig. 6a–f). Western blot data showed a significant suppression of profibrotic targets including MRTFB, RUNX2 (only observed a trend in reduction), COL1A1 and SM22 (protein coded by *TAGLN*) (Fig. 5g–l).

### ACLP regulates cardiac fibroblast activation via the RUNX2 signaling pathway.

**Figure 6:**
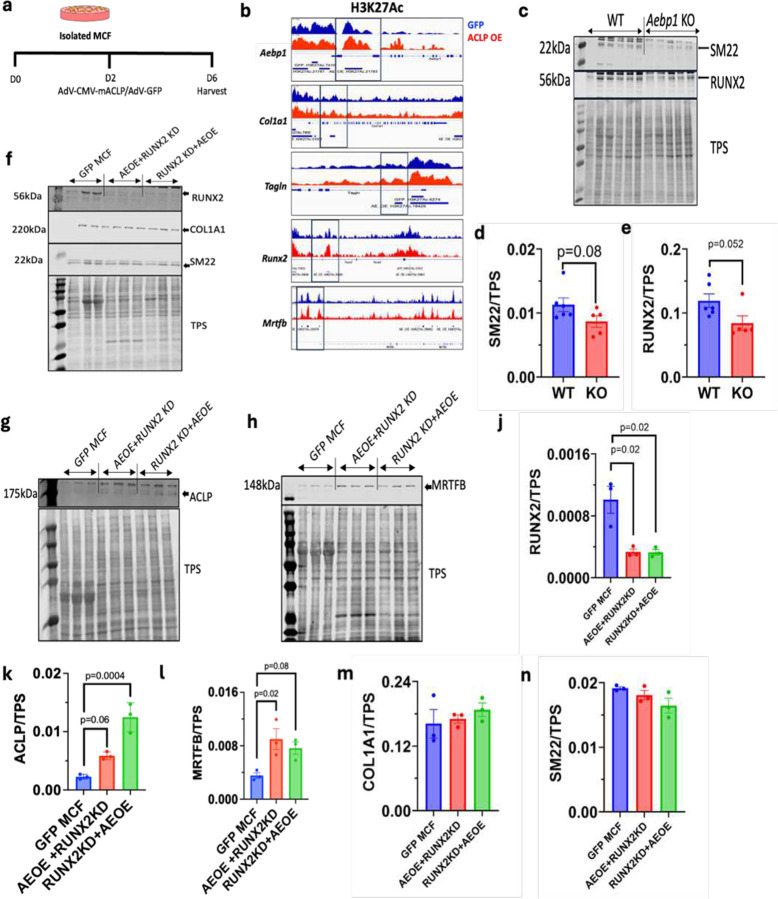
ACLP regulates cardiac fibroblast activation via the RUNX2 signaling pathway. a, Schematic of cell culture using isolated mouse cardiac fibroblasts (MCF). b, CUT&RUN data on mouse cardiac fibroblasts (MCF) using H3K27Ac. c-e, Western blot image and quantification of SM22 and RUNX2 in WT vs fibroblast-specific Aebp1 knockout (KO) MCF (n=6). f-n, Western blot image and quantification of RUNX2, ACLP, MRTFB, COL1A1 and SM22 in WT GFP, RUNX2 KD following and prior to ACLP OE respectively (n=3 each). p-value: Two-tailed unpaired t-test (d&e), One way ANOVA (g-k).

To understand the transcriptional regulation of ACLP in fibroblast activation, we performed cleavage under targets and release using nuclease (CUT&RUN) analysis using H3K27Ac antibody, in mouse cardiac fibroblasts (MCF). H3K27Ac is a critical epigenetic modification associated with active enhancers and gene transcription in the genome^38^. Analysis of H3K27Ac enrichment revealed potential transcriptional activation of pro-fibrotic genes such as *Col1a1* and *Tagln2*, as well as pro-fibrotic transcription factors, *Runx2* and *Mrtfb* following ACLP OE compared to GFP control. Notably, peaks in these loci were unique to ACLP OE and did not reach the threshold for peak calling in GFP controls (Fig. 6a–b). Consistent with the findings from HCF (Fig. 5i–l), fibroblast-specific *Aebp1* KO mice showed a significant reduction in SM22 and RUNX2 reiterating the role of RUNX2 in mediating fibroblast activation (Fig. 6c–e). To further delineate the role of RUNX2 in ACLP-driven fibroblast activation, we performed RUNX2 KD using shRNA (AdV-U6-shRNA-m*Runx2*) either prior to or following ACLP OE in MCF. RUNX2 expression was significantly reduced under both conditions, confirming effective KD. MRTFB levels remained elevated compared to GFP control, indicating either an independent ACLP-MRTFB signaling pathway that is differed from ACLP-RUNX2 pathway or that MRTFB could be upstream of RUNX2. RUNX2 KD suppressed COL1A1 and SM22 expression consistent with previous studies^23,39^, indicating that RUNX2 is potentially downstream of ACLP and regulates COL1A1 and SM22 expression (Fig. 6f–n). Overall, our data demonstrated that ACLP regulates critical pro-fibrotic transcription factors and gene expression programs that promote fibroblast activation. We showed here that RUNX2 was part of the ACLP signaling pathway in addition to previously reported NFkb and Akt signaling pathways^10,35,36^ underscoring the multifaceted role of ACLP in myocardial fibrosis.

### Discussion.

Although fibroblast activation following cardiac injury is an essential repair mechanism to prevent cardiac rupture, persistent activation results in excessive collagen deposition and cross-linking, driving maladaptive remodeling and progression to HF^30,31^. Therefore, identifying therapeutic targets capable of attenuating fibroblast activation is of critical importance. ACLP/AEBP1 has previously been implicated in fibroblast proliferative and activation across multiple organs, including the liver, lung, adipose tissue and kidney, where tissue-specific KD attenuates fibrosis in preclinical mouse models^9,10,12^. Elevated *AEBP1* expression has been reported in HF patients^13^ and in vitro studies have demonstrated its ability to activate cardiac fibroblasts. However, the role of *AEBP1* in vivo following cardiac injury has not been explored.

In this study, we reported a significant increase in *AEBP1* expression in advanced HF patients with extensive myocardial fibrosis. ACLP/AEBP1 localized to infarct and peri-infarct regions in both advanced HF patients and mouse models of MI. Due to increased expression of *AEBP1* in activated fibroblasts following cardiac injury, we used a periostin-specific KO of *Aebp1*. Our data indicates that *Aebp1* deletion or KD after acute MI significantly improved cardiac function compared to untreated WT controls. In contrast, *Aebp1* KO initiated after HF development (EF<35%) did not fully rescue the phenotype, although partial improvement was observed. Whether an intervention earlier in the time course of fibrosis development or a longer duration intervention may have been more effective in reversing the phenotype requires further investigation.

Given the reversible potential of diffuse interstitial fibrosis compared to replacement fibrosis post-MI, we have shown that *Aebp1* KO reduced cardiac hypertrophy and myocardial interstitial fibrosis post infusion of AngII/PE^40,41^. Previous studies have shown that reducing interstitial fibrosis indirectly mitigates cardiomyocyte hypertrophy^1^, which may explain the reduced hypertrophy observed following *Aebp1* inhibition.

Analysis of publicly available CITE-Seq datasets revealed a multi-fold increase in AEBP1 expression in fibroblasts from diseased human hearts, spanning acute MI and chronic HF (ischemic and non-ischemic cardiomyopathy)^42^ (Supplemental Fig. 7). We also demonstrated that ACLP OE in non-failing donor heart slices induced significant remodeling of cardiomyocyte structure and shape whereas *AEBP1* KD in HF myocardium resulted in reduced myocardial fibrosis and reversed myocardial structure and shape similar to non-failing myocardium. These findings support the potential reversibility of fibrosis through *AEBP1* inhibition.

AEBP1 has been shown to function as a transcriptional activator or repressor either by binding to the DNA or by interacting with other transcription factors using its carboxypeptidase domain^43,44^. AEBP1 has been implicated as regulator of the MAPK/ERK and NFκβ signaling pathways^45,46^. ACLP is a non-nuclear isoform that contains a collagen-binding domain and can bind to collagen to stabilize the ECM. AEBP1 protein has been suggested to be translated from alternative mRNA splicing^47,48^. However, the mechanism in which the cells decide to make one isoform or the other is unclear and needs further investigation.

To investigate the mechanistic involvement of ACLP mediated fibroblast activation, we identified pro-fibrotic transcription factors (*Mrtfb* and *Runx2*) and genes (*Tagln* and *Col1a1*) as actively transcribed targets following ACLP OE. Consistent with prior evidence that ACLP enhanced MRTFA activity, our data suggested that ACLP also promotes MRTFB signaling in cardiac fibroblasts^29^. RUNX2, a well-studied transcription factor also regulates fibroblast activation in multiple organs, including the heart^39^. We showed that RUNX2 is a critical signaling transcription factor potentially downstream of ACLP mediated fibroblast activation, whereas MRTFB might have either a divergent pathway from RUNX2 or could be upstream of RUNX2. Furthermore, RUNX2 KD suppressed COL1A1 and SM22 both prior to and following ACLP overexpression, indicating that RUNX2 directly regulates COL1A1 and SM22 expression. Importantly, *Aebp1* KO mice also exhibited a significant downregulation of RUNX2 and reduced SM22, corroborating our in vitro findings.

Our results expand upon previously reported NF-κβ^20^ and PI3K-AKT^49^ signaling pathways by demonstrating that ACLP/AEBP1 also regulates MRTFB and RUNX2 transcriptional activity in cardiac fibroblasts. Inhibition of *AEBP1,* suppressed fibroblast activation by attenuating these pathways and their downstream targets (Supplemental Fig. 8). Although previous studies have suggested a link between *AEBP1* and *RUNX2*^50^, our finding reveals a novel mechanism of ACLP/AEBP1 mediated RUNX2 regulation in cardiac fibroblast activation.

In summary, we identify AEBP1/ACLP as a central regulator of cardiac fibroblast activation and propose AEBP1 inhibition as a promising therapeutic strategy to mitigate fibrosis and adverse remodeling following cardiac injury and stress.

## Methods

### Animals and animal care.

All animal studies were performed in accordance with the Institutional Animal Care and Use Committee (IACUC). All procedures involving animals were approved by the Animal Care and Use Committee of the University of Utah and complied with the American Physiological Society’s *Guiding Principles in the Care and Use of Animals* and the UK Animals (Scientific Procedures) Act 1986 guidelines. The mice were housed in 12 h dark/light cycle at 70°F and 40% humidity. AEBP1 floxed mice as previously described^15^ were crossed with Periostin-MerCreMer (Strain #029645). Heterozygous matings were set up to obtain KO mice and all mice used in this study received 3 consecutive intraperitoneal tamoxifen injection (40 kg/g/d) at 8 weeks of age. Both genders were used in this study.

### Ethical approval for human samples:

Cardiac tissue samples from advanced HF patients were used in this study. Control tissue was acquired from non-failing donors (based on echocardiography data) that was rejected for transplant for non-cardiac reasons such as infection, size mismatch, etc. Tissue was acquired from centers included in the U.T.A.H (Utah Transplantation Affiliated Hospitals) Cardiac Transplant Program (University of Utah Health Science Center and Intermountain Medical Center). The institutional review board of each of the two institutions approved the study and all patients provided informed consent.

### Masson’s trichrome/ Fibrosis analysis for Human Tissue.

Human myocardial tissue used for this experiment was fixed with 4% paraformaldehyde (PFA) followed by paraffin embedding. 8μm thick sections were cut and stained with Trichrome for fibrosis analysis using the Dako automated special strainer. The slides for fibrosis analysis were scanned under 20x and analyzed using Aperio Image Scope software (version 12.3.2.8013) (using the colocalization v9 algorithm) as previously mentioned^51^. A ratio of the total stained area to the collagen-stained area was reported.

### RNA Extraction and qRT-PCR.

Human myocardial tissue, HCF and MCF were used for this experiment. miRNeasy Mini kit (Qiagen) was used for RNA extraction. The extracted RNA was used for total RNA sequencing (RNA Seq). Agilent RNA Screen Tape Assay was used for QC experiments. Illumina TruSeq Stranded RNA kit was used for library preparation and Ribo-Zero Gold was used to remove rRNA and the sequencing performed on an Illumina HiSeq 2500 with 50bp single-end reads.

### RNA Seq analysis.

RNA Seq analysis was performed with the High-Throughput Genomics and Bioinformatics Analysis Shared Resource at the Huntsman Cancer Institute at the University of Utah. mm10, M_musculus_Dec_2011, GRCm38 genome build was used for sequence alignment for mouse samples and Ensemble90 was used for human genome alignment. Sample outliers were checked by summarizing the output files from cutadapt, FastQC, Picard CollectRnaSeqMetrics, STAR, and feature counts by using the previously described method42. 5% false discovery rate (FDR) with DeSeq2 (version 1.24.0) was used to identify differentially expressed genes43. Genes were filtered using the following criteria: adjusted p-value < 0.05, absolute log2 fold change > 0.585 and normalized base mean >3043. Heat maps were produced using the R library p heatmap. KEGG44 and Ingenuity pathway analysis software was used for gene ontology analysis.

### Human single nuclei isolation, snRNA sequencing and analysis.

n=14 non-failing donor left ventricular myocardium and n=13 HF left ventricular myocardium was used for this study. Nuclei extraction, library preparation, sequencing protocol and analysis for these sample sets are the same as reported in our previous publication^52^. CITE-Seq data were obtained from Amrute et. al^42^

### Immunohistochemistry (IHC).

Both mice and human heart tissues were used for this study. Cardiac tissues were embedded in OCT solution after PFA fixation and sectioned at 100μm thickness and staining was performed as previously described^51^. Antibodies used in this experiment are listed in Table 1. Secondary anti-mouse or anti-rabbit were used appropriately. Samples were imaged in a Leica confocal microscope using 63x oil lens and at a pixel size of 0.2 mm. All images for each protein were acquired using the same laser settings across samples and processed using Fiji software.

### Image Analysis.

Two-dimensional confocal images (1024×1024 pixels, pixel size 0.18×0.18 μm^2^) were noise-filtered and deconvolved. Subsequently, local histogram-based thresholds were applied to separate noise from background in all channels as described recently^53,54^. A local mean filter with a box size fitting to the expected signal cluster size was applied and the result subtracted from the original image. The standard deviation *s* and image mode *m* of the resulting image were used to determine the threshold *t* determined with *t* = *c* • *s* + *m*. The factor *c* was chosen 2 for DAPI, 1 for WGA, 1 for vimentin, and 1 for periostin. Box sizes of the mean filter were 50×50 μm^2^ for DAPI, 10×10 μm^2^ for vimentin and periostin. For WGA, two different box sizes (2.5×2.5 μm^2^ and 150×150 μm^2^) were used and the resulting segmented images merged to account for small structures, such as cell membranes, but also larger structures, such as fibrotic infarct regions. The obtained binary images of the signal were then median filtered with radius 1. The fraction of the signal was calculated by dividing the number of positive pixels by the total number of pixels in the image. Cell geometry was analyzed after creating cell segments based on a watershed transform^54^. ECM% was calculated using the WGA data. Cell surface area was calculated by multiplying cell length and cell width using ImageJ/Fiji. Cross sectional images were not included in this measurement.

### Human cardiac fibroblast (HCF) culture – TGFβ stimulation, overexpression (AdV-CMV-hAEBP1-GFP) and knockdown (AdV-pEQU6-hAEBP1-shRNA).

HCF (PromoCell #c-12375) was cultured in HCF media (PromoCell #C-23010) on 12-well plates with 50,000 cells/well for 48h. Cells were stimulated with 10 ng/ml TGFb for 48h with media change every 24h. Viral transduction (ACLP OE or KD) was performed at 250 MOI for 72h before or after stimulation. The cells were infected with virus for 24h and then the media changed every 24h. Cells were harvested at the end of the experiment using cell scrapper. Vector sequences are available in the supplement.

### Mouse cardiac fibroblast isolation and cell culture.

MCFs were isolated as previously described^55^. Cells were cultured in DMEM/F12 with antibiotics and 10% FBS on a 10 cm petri dish and media exchange was performed every 24h. TGFb stimulation and viral transduction were same as described above for HCF. Vectors specific to mouse AEBP1 were used for this experiment. AdV-GFP was used as control, AdV-CMV-m*Aebp1*-Ef1a-GFP (overexpressing the full-length ACLP) was used for ACLP OE and AdV-pEQU6-sh-m*Aebp1*-GFP was used for AEBP1 KD. AdV-pEQU6-m*Runx2*-shRNA-GFP was used for RUNX2 KD. Vector sequences are available in the supplement.

### CUT&RUN experiment and analysis.

Isolated primary mouse cardiac fibroblasts (ACLP OE and GFP control) were used for this experiment. CUT&RUN kit from Cell signaling (86652P) was used and manufacturer’s instructions were followed. 200,000 cells/reaction was used and DNA purification kit from cell signaling was used to extract DNA (14209S). Samples were sequenced at 25M reads/sample and data analyzed using integrated genome viewer (IGV).

### Protein Extraction.

HCF/MCF were lysed in 1X RIPA buffer (Cell Signaling Technology #9806S) containing 2X protease and phosphatase inhibitor (Thermo Scientific #78440) and was allowed to sit for 20 min. 5 μl of 100 mmol PMSF was added to the homogenate and allowed to rotate for 30 min at 4°C, followed by 10 min of centrifugation at 4°C at 18000 rpm. Supernatant was transferred to a new tube and Pierce BCA Protein Assay kit (Thermo Scientific, #23225) was used for protein estimation. Equal volume of 2X Laemmli buffer with 10% DTT was added to the supernatant and boiled for 10 min at 98°C. Tissue protein extraction was performed as previously described^51^.

### Western Blotting.

Using 30 μg of protein, gels were run at constant volts (25V/gel) and then transferred to a nitrocellulose membrane at a constant current (350 mA) for 1h. A total protein stain (TPS) was then performed, and the membrane was scanned in accordance to Licor Revert 700 TPS procedure. Membranes were then blocked with Odyssey Blocking Buffer (LiCor #927–50000) and subsequently probed with primary antibodies overnight. The antibodies used and their concentration are listed in Table 1. Following primary probing, blots were washed with 1X TBS-tween thrice and incubated with secondary antibodies (anti-mouse or anti-rabbit 1:10,000) for 30 min in the dark. Prior to scanning blots with Odyssey Infrared Imager an additional three washes with 1X TBS-tween were performed. Image Studio Lite software version 5.2 was used to analyze the blots. Each gel had its own TPS and the proteins were normalized to its respective TPS. Outliers, if identified by GraphPad prism were removed.

### Mouse myocardial infarction (MI) model.

Male and female 12-week-old C57BL6J mice were used for this study. Mice were anesthetized with 3% isoflurane mixed with oxygen, fur removed using nair and intubated. A thoracotomy was performed and the muscle between the 4th and 5th intercostal was cut to expose the heart. A 6–0 proline suture was used to ligate the proximal left anterior descending coronary artery. The muscle and skin were sutured back and the mice allowed to recover in a heating pad. Analgesics were administered as per the approved protocol.

### AngII+PE systemic fibrosis model.

12-week-old C57BL6J mice were used for this study. Both male and female mice were used for all experiments. Mice were anesthetized with 3% isoflurane mixed with oxygen and the hair removed using nair. Alzet mini-osmotic pump (2004) was prepared as per manufacturers protocol either with saline or with a combination of Angiotensin II (1.5 mg/g/day) and Phenylephrine (50 mg/g/day). A small incision was made and the pumps inserted onto the back of the mice and the skin sutured. Mini-osmotic pump weights were taken before implantation and after the end of the study. Analgesics were administered as per the approved protocol.

### Retro-orbital injections.

4 days post MI mice and 2 weeks post AngII+PE administered mice were randomly chosen to receive either treatment vector (global KD: AAV9-CMV-turboGFP-mi*Aebp*1, fibroblast-specific KD: AAV9-Postn-GFP-m*Aebp1*-miR30G) or control vector (AAV9-turbo-GFP or AAV9-Postn-GFP-miR30G control). N = 5 mice were used in each group, and a total of 2 groups were used for the MI study (treated and untreated) and 3 groups for the AngII+PE study (control saline, untreated with drug combination and treated with drug combination). A concentration of 3.1e12 VG kg−1 was used for each injection and the mice were serially echoed for 4 weeks.

### Enzyme Linked Immunosorbent Assay (ELISA).

AEBP1 ELISA kit (Biorbyt orb437774) was used to perform ELISA on cell culture supernatant and mouse serum. Manufacturers protocol was followed.

### Echocardiographic analysis.

Mice were anesthetized with 1.5% Isoflurane (Vet One, NDC13985-046-60) during echocardiography. Echocardiographic images were taken on the Vivo system. Echoes were performed serially and 2D long-axis and short-axis views were obtained and used for analysis using Vivo strain software (version 3.1.1). Two consecutive cardiac cycles were used for all the measurements. Limb leads were used to record an electrocardiogram (ECG)^51^.

### Myocardial tissue culture.

Human HF and non-failing donor hearts were used for this study. Myocardial tissues were sectioned at approximately to a size of 1mm × 1mm × 0.3mm and mounted onto the chambers. All sections were electrically stimulated at 50–80mA/1ms at a frequency of 0.5 Hz as previously published^33,56^. M199 media with ITS (Thermo Scientific #41400045) and antibiotics (Corning #30–004-CI) was used to culture the slices and media exchange was performed every 48h. The tissue was allowed to recover from the sectioning and processing for up to 7 days and random slices were chosen to receive either GFP (Ad-CMV-*GFP*), *AEBP1* OE (Ad-CMV-*AEBP1*-GFP) or AEBP1 KD (Ad-U6-sh*AEBP1*-*GFP*). 1×10^12^ vp/ml was added to each slice and media exchange was performed 24h after viral transduction. At 2 weeks post viral treatment, tissue slices were fixed for 10 min with 2% PFA and frozen in OCT for further processing.

### Statistics and reproducibility.

All data were summarized as mean±SEM. GraphPad Prism (version8.2.1) was used for all statistical analyses. Unpaired two-tailed Student’s and two-tailed Mann-Whitney test were used for all analyses with two groups and mentioned appropriately. Multiple t-test and one-way ANOVA were applied for longitudinal echocardiographic data and while comparing multiple groups, respectively. All experiments were repeated independently (at least three biological replicates, and three technical replicates wherever applicable) with reproducible results. Samples used for western blot quantification were from the same gel.

## Supplementary Material

1

Supplementary Files

This is a list of supplementary files associated with this preprint. Click to download.

- SupplementalFiguresandlegends1.pdf

## Figures and Tables

**Table 1:** List of antibodies and fluorophores used.

Antibody	Host	Concentration	Identifier	Source
**Western blot**				
MKL2/MRTFB	Rb	1:500	14613	Cell Signaling Technology
Transgelin 2 (E3R3I) (SM22)	Rb	1:500	62567	Cell Signaling Technology
Collagen1a1	Rb	1:100	NB600–408	Novus Biologicals
RUNX2	Rb	1:1000	ab-192256	Abcam
ACLP/AEBP1	Ms	1:100	sc-271374	Santa Cruz Biotechnology
Anti-mouse 680	Dk	1:10000	926–68072	LiCOR Biosciences
Anti-rabbit 800	Dk	1:10000	926–32213	LiCOR Biosciences
**IHC**				
4',6-diamidino-2-phenylindole (DAPI)		1:1000	D3571	Thermo Fisher Scientific
Wheat germ agglutinin		1:1000	W32464	Thermo Disher Scientific
AEBP1	Ms	1:100	sc-271374	Santa Cruz Biotechnology
Periostin/OSF-2 antibody	Rb	1:100	30042	Novus Biologicals
Vimentin (RV202)	Ms	1:100	sc-32322	Santa Cruz Biotechnology
MyosinIIb	Rb	1:100	8824S	Cell Signaling Technology

## Data Availability

Source data can be included in the manuscript when requested. RNA sequencing data will be uploaded to GEO upon acceptance of the manuscript.
