# Supplementary material for: Adipocyte Enhancer Binding Protein 1 (AEBP1) Inhibition as a Potential Anti-Fibrotic Therapy in Heart Failure
Source: Res Sq. 2026 May 26:rs.3.rs-3390276. Preprint. [Version 2] doi: 10.21203/rs.3.rs-3390276/v2 (PMC13232471; doi:10.21203/rs.3.rs-3390276/v2)
Supplement: 1 [file NIHPPRS3390276V2-supplement-1.pdf]

Supplemental Figures and legends.

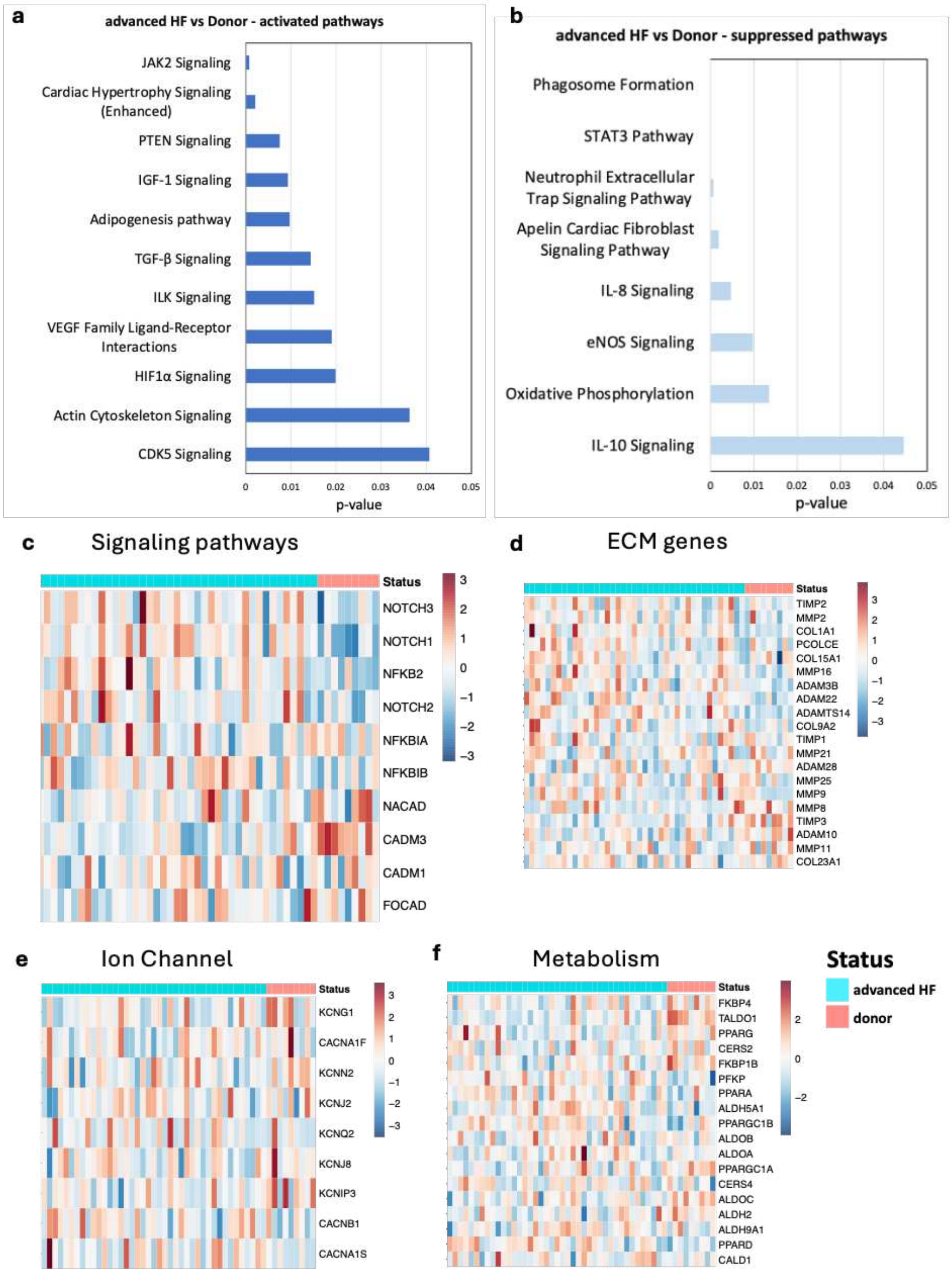

Supplemental Fig. 1: a-b, Ingenuity pathway analysis showing activated and suppressed pathways in advanced HF myocardium compared to non-failing donor myocardium (n=9 donor,

n=41 HF). **c-f,** Heat map of genes involved in cell signaling pathways, ECM organization, Ion channel physiology, and metabolism respectively (n=9 donor, n=41 HF).

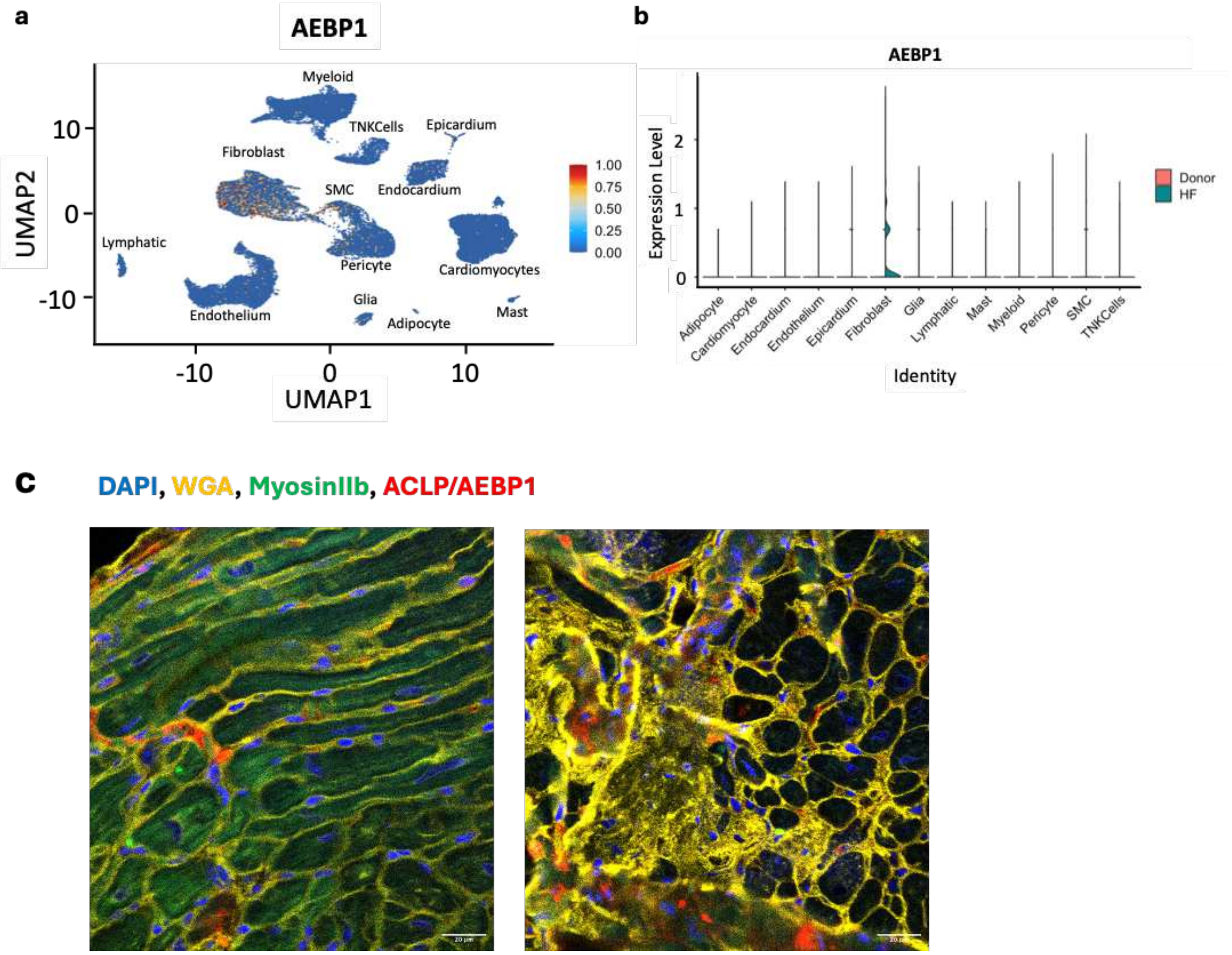

Supplemental Fig. 2: a, Single-nuclear RNA sequencing (snRNA seq) data showing *AEBP1* expression in human non-failing donor myocardium across different cell types (n=14 donor). **b,** Violin plot of snRNA seq showing differential expression of *AEBP1* between donor and HF myocardium across cell types (n=14 donor, n=13 HF). **c.** Representative immunohistochemistry image on mouse myocardium 4days post-MI (n=3).

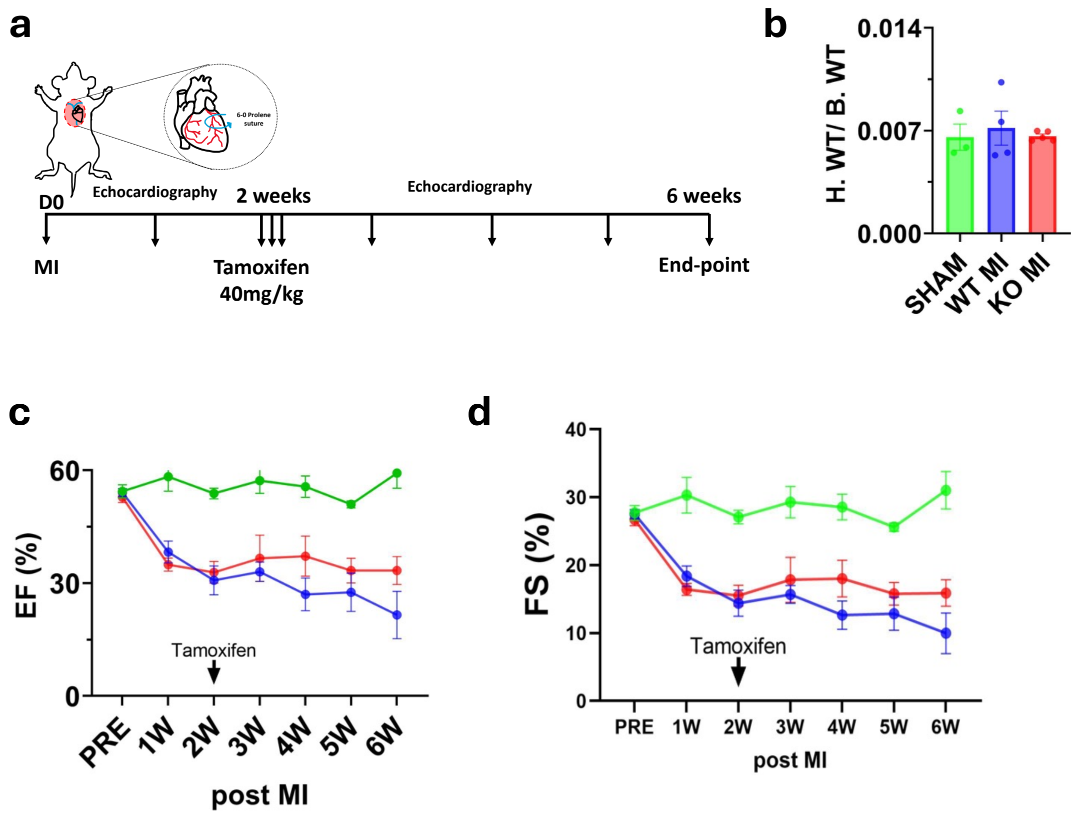

Supplemental Fig. 3: a, Schematic of heart failure induction and fibroblast-specific *Aebp1* KO. **b,** Heart weight to body weight ratio (n=3 Sham, n=4 WT MI, n=5 KO MI). **c-d,** Ejection fraction (EF%) and fractional shortening (FS%) data (n=3 Sham, n=4 WT MI, n=5 KO MI).

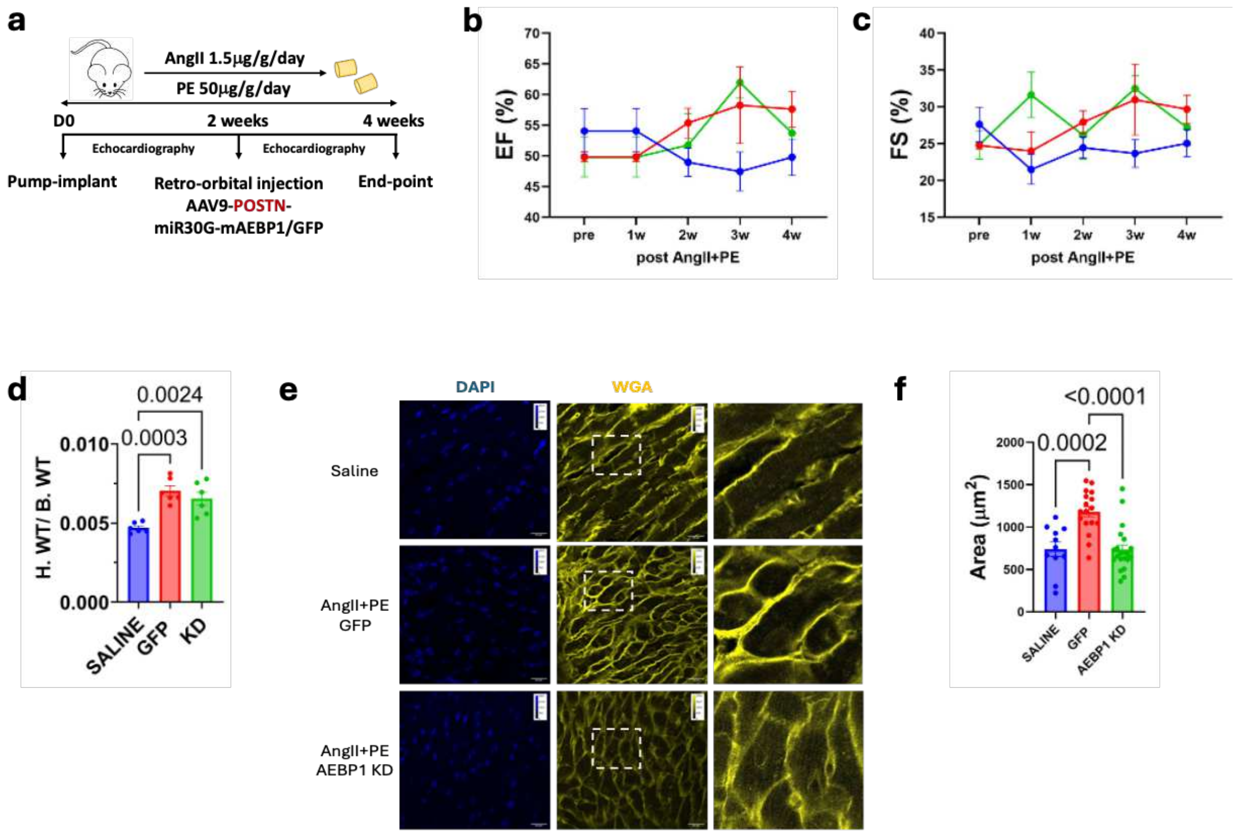

Supplemental Fig. 4: a, Schematic of AngII/PE infusion and fibroblast-specific *Aebp1* KD using AAV9. **b-c,** Ejection fraction (EF%) and fractional shortening (FS%) data (n=6 each). **d,** Heart weight to body weight ratio (n=6 each). **e,** Representative immunohistochemistry data (n=6 each). **f,** Cell surface area quantification (n=6 mice, multiple images/animal). p-value: One way ANOVA.

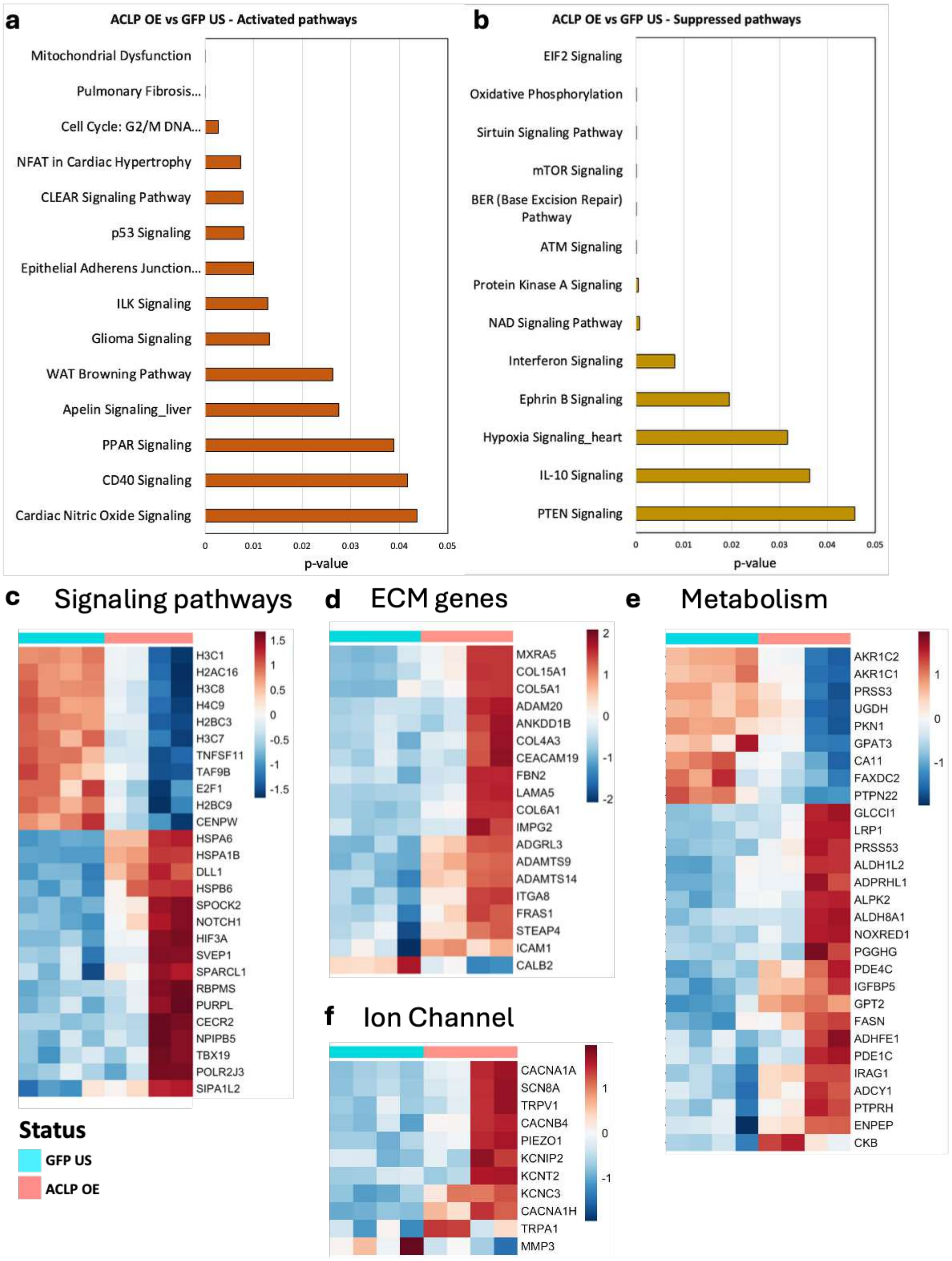

Supplemental Fig.5: a-b, Ingenuity pathway analysis of RNA sequencing data from HCF following ACLP overexpression compared to unstimulated (US – no TGF-beta) GFP (n=4 each). **c-f,** Heat maps of genes involved in cell signaling, ECM organization, metabolism and ion channel signaling respectively (n=4 each).

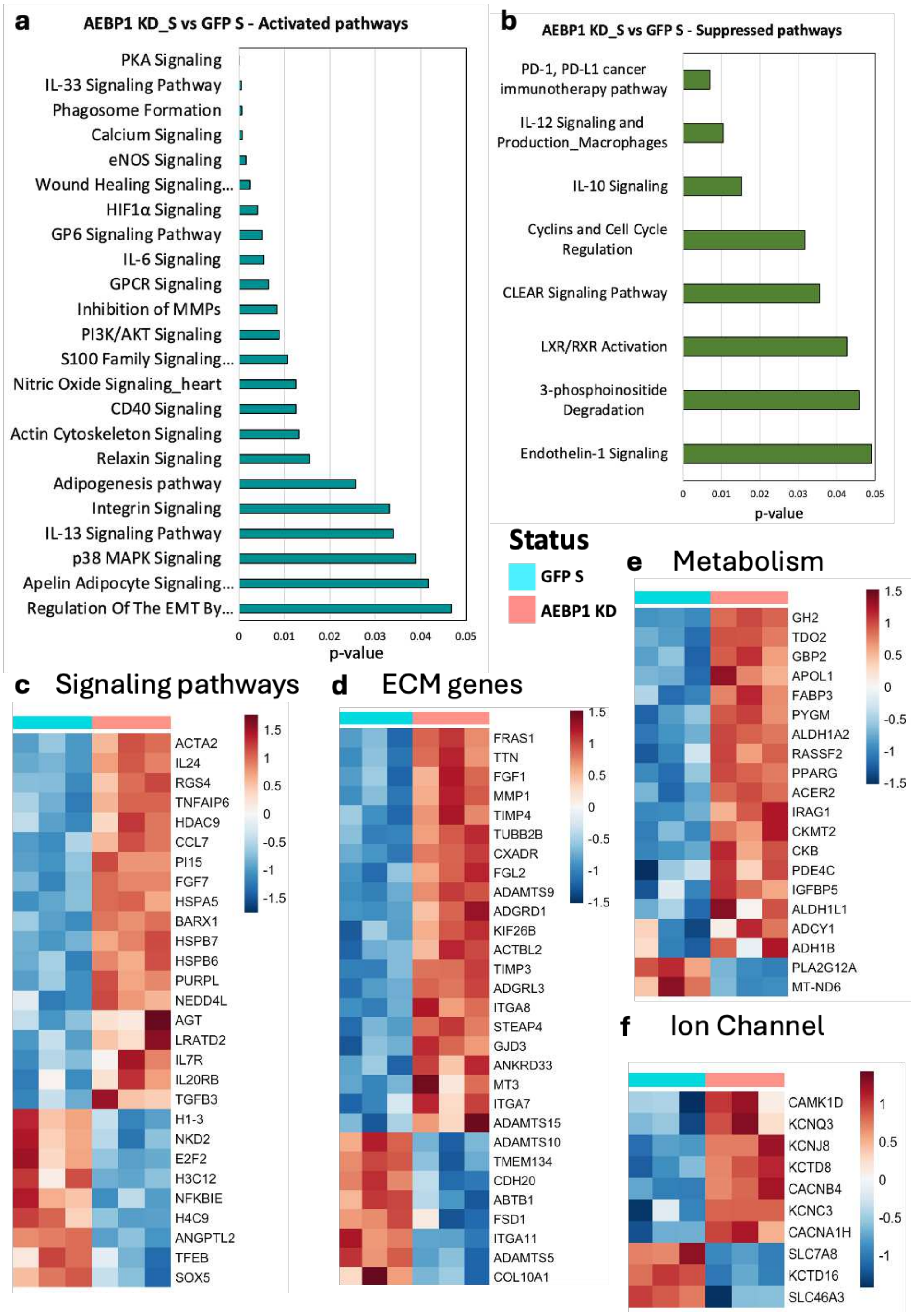

Supplemental Fig.6: a-b, Ingenuity pathway analysis of RNA sequencing data from HCF following *AEBP1* KD compared to GFP S (S - TGFβ stimulated) (n=3 each). **c-f,** Heat maps of genes involved in cell signaling, ECM organization, metabolism and ion channel signaling respectively (n=3 each).

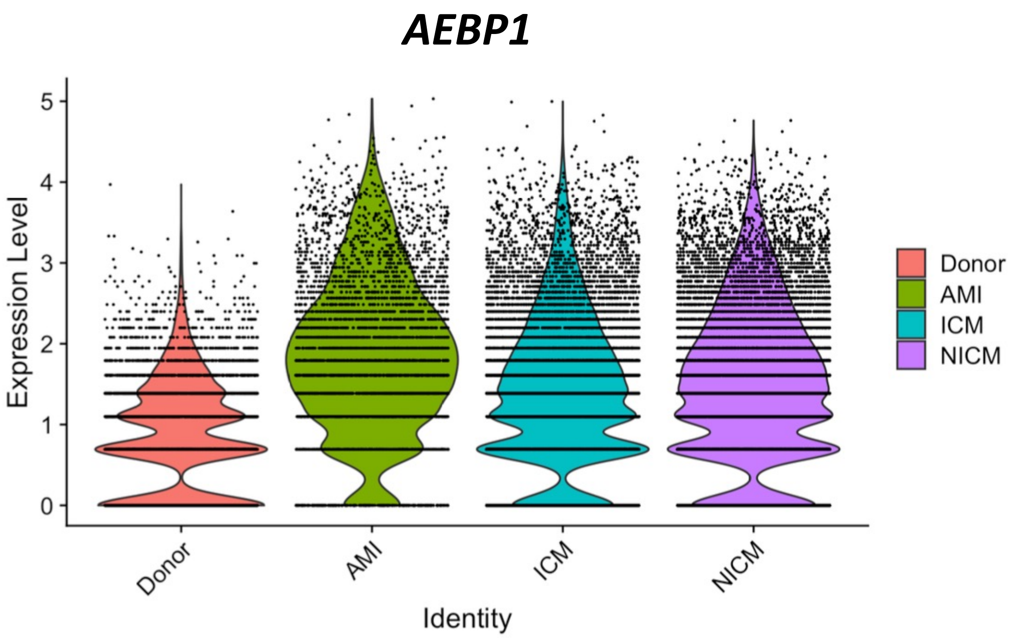

Supplemental Fig. 7: CITE-Seq data from non-failing donor (n=6), patient with acute myocardial infarction (AMI, n=4) and chronic HF (ICM- ischemic cardiomyopathy (n=6) and NICM- non-ischemic cardiomyopathy (n=6)) showing *AEBP1* expression in fibroblasts.

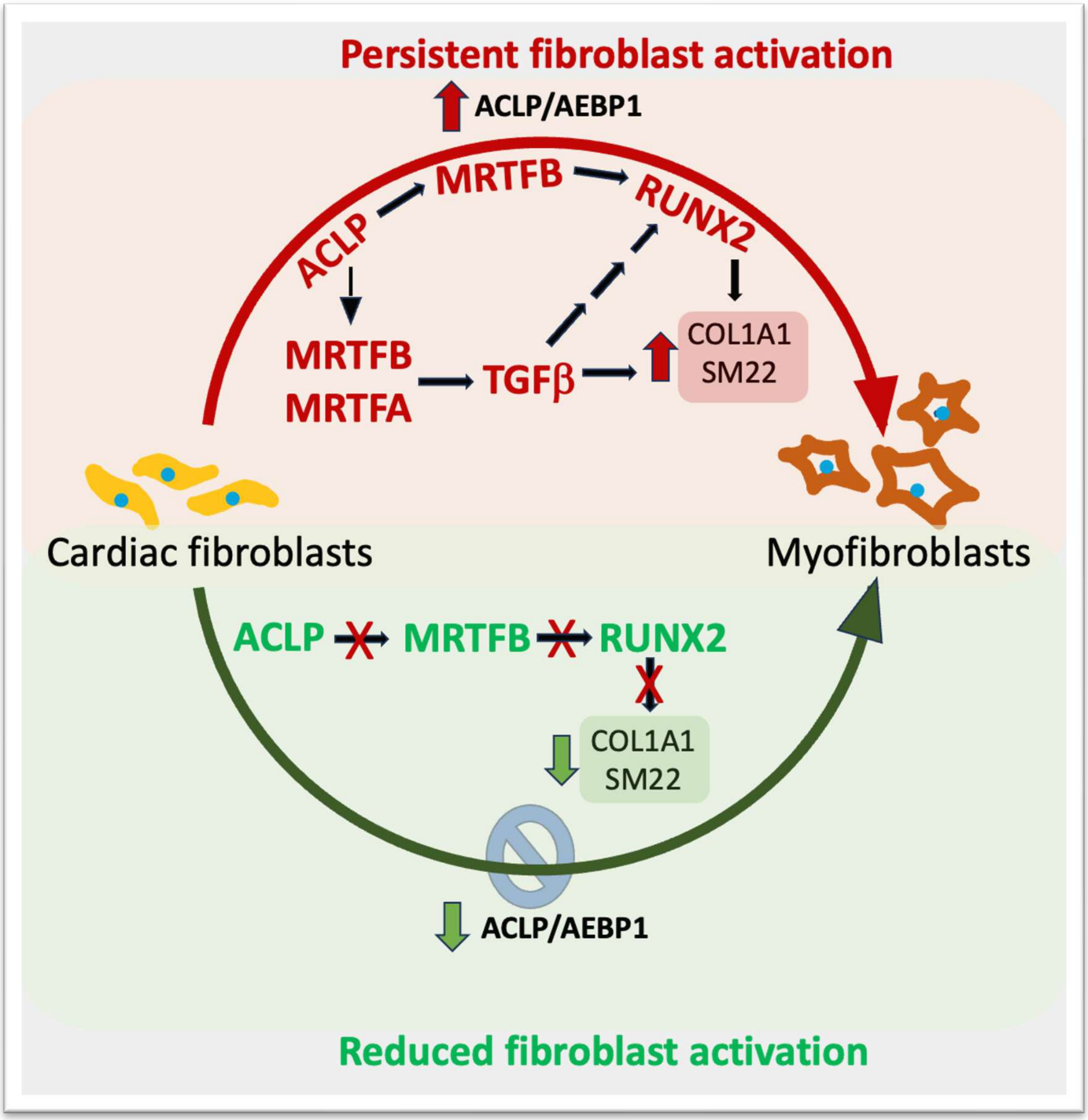

Supplemental Fig. 8: Elevated ACLP/AEBP1 expression promotes cardiac fibroblast activation through MRTFB- and RUNX2-dependent signaling pathway. RUNX2 activation leads to increased SM22 and COL1A1 production, whereas AEBP1 knockdown (KD) suppressed fibroblast activation by inhibiting both RUNX2 and MRTFB. RUNX2 KD suppressed SM22 and COL1A1 secretion without altering MRTFB levels, suggesting RUNX2 functions downstream of ACLP/AEBP1 signaling. These finding supports the existence of an ACLP-RUNX2, ACLP-MRTFB or integrated ACLP-MRTFB-RUNX2 signaling cascade driving cardiac fibroblast

activation. Overall, the data identify AEBP1 inhibition as a potential therapeutic strategy for cardiac fibrosis attenuation.

Vector maps

Human in vitro and ex vivo AEBP1 KD vector:

pEQU6-hAEBP1-shRNA vector

hAEBP1-shRNA: cacgaggcctcaagatctat

MCF in vitro ACLP OE:

pEntCMV-mAEBP1-HA

```
1141 atgg ctccagtgcg caccgcatcc ctgctctgcg gcctcctggc actgctgacg
1201 ctgtgccctg aggggaaccc acagacggtg ctgacggacg acgagatcga ggagttcctc
1261 gaaggcttcc tttcggagtt ggagacccag tccccgcccc gggaagacga cgtggaagtc
1321 cagccgcttc ccgaacccac ccagcgtccc cgcaaatcca aggcaggggg caagcagcgg
1381 gcagatgtag aagtccctcc agaaaaaaac aaagacaaag agaagaaagg aaagaaggac
1441 aaaggcccca aagccacaaa acccctggag ggctctacca ggcccaccaa gaaaccaaag
1501 gagaagccac ccaaggccac caagaagccc aaggagaaac cacccaaggc caccaagaag
1561 cccaaggaga agccacccaa ggccaccaag aagcctaagg agaagccacc caaggccact
1621 aagaggccct cggcaggaaa gaagttctca actgtggccc ccttggaaac gctggatcgg
1681 ttactcccct caccctccaa ccccagcgcc caggagctac cgcagaagag agacacaccc
1741 ttcccaaatg cctggcaagg tcaaggagaa gagacccagg tggaggccaa gcagccccgg
1801 ccagagccag aggaggagac tgagatgccc acactggact acaatgacca gatagagaag
1861 gaggattacg aggattttga gtacatccgt cgccagaagc agcccaggcc aacacccagc
1921 aggaggaggc tctggccaga gcgccctgag gagaagactg aagagccaga ggaaaggaag
1981 gaagtcgagc cacctctgaa gcccctgctg cctccggact atggggatag ctacgtgatc
2041 cccaactatg atgacttgga ctattatttc cccaccctc caccgcagaa gcctgatgtt
2101 ggacaagagg tggatgagga aaaggaagag atgaagaagc ccaaaaagga gggtagtagc
2161 cccaaggagg acacagagga caagtggacc gtggagaaaa acaaggacca caaagggccc
2221 cggaagggtg aggagctgga ggaggagtgg gcgccagtgg agaaaatcaa gtgcccacct
2281 attgggatgg agtcacaccg cattgaggac aaccagatcc gtgcctcctc catgctgcgc
2341 cacggcctcg gagcccagcg gggccggctc aacatgcagg ctggtgccaa tgaagatgac
2401 tactatgacg gggcatggtg tgctgaggac gagtcgcaga cccagtggat cgaggtggac
2461 acccgaagga caactcggtt cacgggcgtc atcactcagg gccgtgactc cagcatccat
2521 gacgacttcg tgactacctt ctttgtgggc ttcagcaatg acagccagac ctgggtgatg
2581 tacaccaatg gctacgagga aatgaccttc tatggaaatg tggacaagga cacacctgtg
2641 ctgagcgagc tccctgagcc agttgtggcc cgtttcatcc gcatctatcc actcacctgg
2701 aacggtagcc tgtgcatgcg cctggaggtg ctaggctgcc ccgtgacccc tgtctacagc
2761 tactacgcac agaatgaggt ggtaactact gacagcctgg acttccggca ccacagctac
2821 aaggacatgc gccagctgat gaaggctgtc aatgaggagt gccccacaat cactcgcaca
2881 tacagcctgg gcaagagttc acgagggctc aagatctacg caatggaaat ctcagacaac
2941 cctggggatc atgaactggg ggagcccgag ttccgctaca cagccgggat ccacggcaat
3001 gaggtgctag gccgagagct cctgctcctg ctcatgcaat acctatgcca ggagtaccgc
3061 gatgggaacc cgagagtgcg caacctggtg caggacacac gcatccacct ggtgccctcg
3121 ctgaaccctg atggctatga ggtggcagcg cagatgggct cagagtttgg gaactgggca
3181 ctggggctgt ggactgagga gggctttgac atcttcgagg acttcccaga tctcaactct
3241 gtgctctggg cagctgagga gaagaaatgg gtcccctaca gggtcccaaa caataacttg
3301 ccaatccctg aacgttacct gtccccagat gccacggtct ccacagaagt ccgggccatt
3361 atttcctgga tggagaagaa cccctttgtg ctgggtgcaa atctgaacgg tggtgagcgg
3421 cttgtgtctt atccctatga catggcccgg acacctagcc aggagcagct gttggccgag
3481 gcactggcag ctgcccgcgg agaagatgat gacggggtgt ctgaggccca ggagactcca
3541 gatcacgcta ttttccgctg gctggccatc tcatttgcct ccgcccatct caccatgacg
3601 gagccctacc ggggagggtg ccaggcccag gactacacca gcggcatggg cattgtcaac
3661 ggggccaagt ggaatcctcg ctctgggact ttcaatgact ttagctacct gcacacaaac
3721 tgtctggagc tctccgtata cctgggctgt gacaagttcc cccacgagag tgagctaccc
3781 cgagaatggg agaacaacaa agaagcgctg ctcaccttca tggagcaggt gcaccgtggc
3841 attaagggtg tggtgacaga tgagcaaggc atccccattg ccaatgccac catctctgtg
3901 agtggcatca accatggtgt gaagacagca agtggaggtg actactggcg cattctgaac
3961 ccgggtgagt accgtgtgac agctcacgca gagggctaca cctcaagtgc caagatctgc
```

4021 aatgtggact acgatattgg ggccactcag tgcaacttca tcctggctcg atccaactgg
4081 aagcgcattc gggagatctt ggctatgaac gggaaccgtc ccattctccg agttgacccc
4141 tcacgaccca tgacccccca gcagcggcgc atgcagcagc gccgtctaca gtaccggctc
4201 cgcatgaggg aacagatgcg actgcgtcgc ctcaattcta ccgcaggccc tgccacaagc
4261 cccactcctg cccttatgcc tccccccttcc cctacaccag ccattacctt gaggccctgg
4321 gaagttctac ccactaccac tgcaggctgg gaggagtcag agactgagac ctatacagaa
4381 gtagtgacag agtttgagac agagtatggg actgacctag aggtggaaga gatagaggag
4441 gaggaggagg aggaggagga agagatggac acaggcctta catttccact cacaacagtg
4501 gagacctaca cagtgaactt tggggacttc tacccatacg atgttccaga ttacgcttga

Global *Aebp1* KD in vivo:

pAAV9-CMV-TurboGFP-siAEBP1
gaaggtatattgctgttgacagtgagcgaacgagggctcaagatctacgctagtgaagccacagatgtagcgtagac
ttgagccctcgtttgcctactgcctcgggaattc

Fibroblast-specific Aebp1 KD in vivo:
pEnt-Postn-TurboRFP-siAEBP1 vector
Yellow=mPostn promoter
Green=siAEBP1
541 gatggaaatt agtacatgat tcttgattta aattctttca agctaacaat cttttttttt
601 tttttaaagt ggcctcagtc aaagacacta aagatcaccg agtcttgcat agagtttcca
661 tttacaggac tagagaaagc tagtggagac acagatcggg tgcggaggta gtgagaagca
721 cttttcctaa gaaggtgcag ggttgactcc aaggcttggc tgggttataa gagttacatg
781 tattatttat tctatatgta agcaactttt gagctcatgt gccatggcaa cctatggacc
841 gcatgttaat atagaagcat tttaaaatta gtgatacaat caagaccaag ggcatcctgc
901 ttatggtttg tgtgcacagg cttacagagt gcagagtccg cgaggagtcc cagggactgc
961 tggagtttga ggttggtttc acagtggtga gtaagcgtgg cagtgtaatg acctcatggt
1021 ctcccgaggc cagataacag agaactgcct ataaatcagc atgccgcggc tagagagaaa
1081 cggccctgtt tctcagacac actatctctc ttcagctaca taatgaacca tttctttctc
1141 agtaatgact tacatctctg ggtcagactt tgcagccctg gaaagtcgga cttcatttc
1201 atgatttccg tcatcttccc gactggtagg aaaattgcag gggtcagtag tgtcagcata
1261 gtttcacaga gctgaagaga aagggccctg tgtggagagc gacttttgat gagagccccg
1321 gaagagagtg tgcccttccg gggatttttt tcccagtctc ttctacaact tcagctagcc
1381 aattgagggg catgtgtctc ttccacataa gctgtggaaa tcacacttta aatgcattgt
1441 acatctatcc aggatttggg ttaaatgccc ctgtgatttc tcttctccgt gttctgctgt
1501 ggagtgattt aagtgcaatc agatcaaacc aggaaagtaa ctgagctcag agacacagag
1561 tgtggtggca gagacagaag gcagagagat ccctaaactc agaatcagct cttttcgcaa
1621 tgtaaaccta tagaagtgaa aaacgggctc accatgattg aaaacaaata ggagacagag
1681 ttcagattgc tcagaaccca ggagatttcc agggacagcc cagggctgct ggtgcttctg
1741 taaggccatc gcaagcttca ggttggccca gcgcccccctc ccacagcctt gctccctccc
1801 acagcccaga gctatataaa ctcagctctc cagagcacag gccagatctc ttcctggacg
1861 gagctcaggg
gaaggtatattgctgttgacagtgagcgaacgagggctcaagatctacgctagtgaagccacagatgtagcgtagat
cttgagccctcgtttgcctactg

Runx2 KD, MCF in vitro:
pEQU6-mRUNX2-shRNA vector: gatgagtctgttt
